# Supplementary material for: Characterizing the role of endocarp a and b cells layers during pod (silique) development in Brassicaceae
Source: Plant Signal Behav. 2024 Jul 29;19(1):2384243. doi: 10.1080/15592324.2024.2384243 (PMC11290770; doi:10.1080/15592324.2024.2384243)
Supplement: v3_Supplementary_Nichol_and_Samuel_2024_manuscript.docx [file KPSB_A_2384243_SM8264.docx]

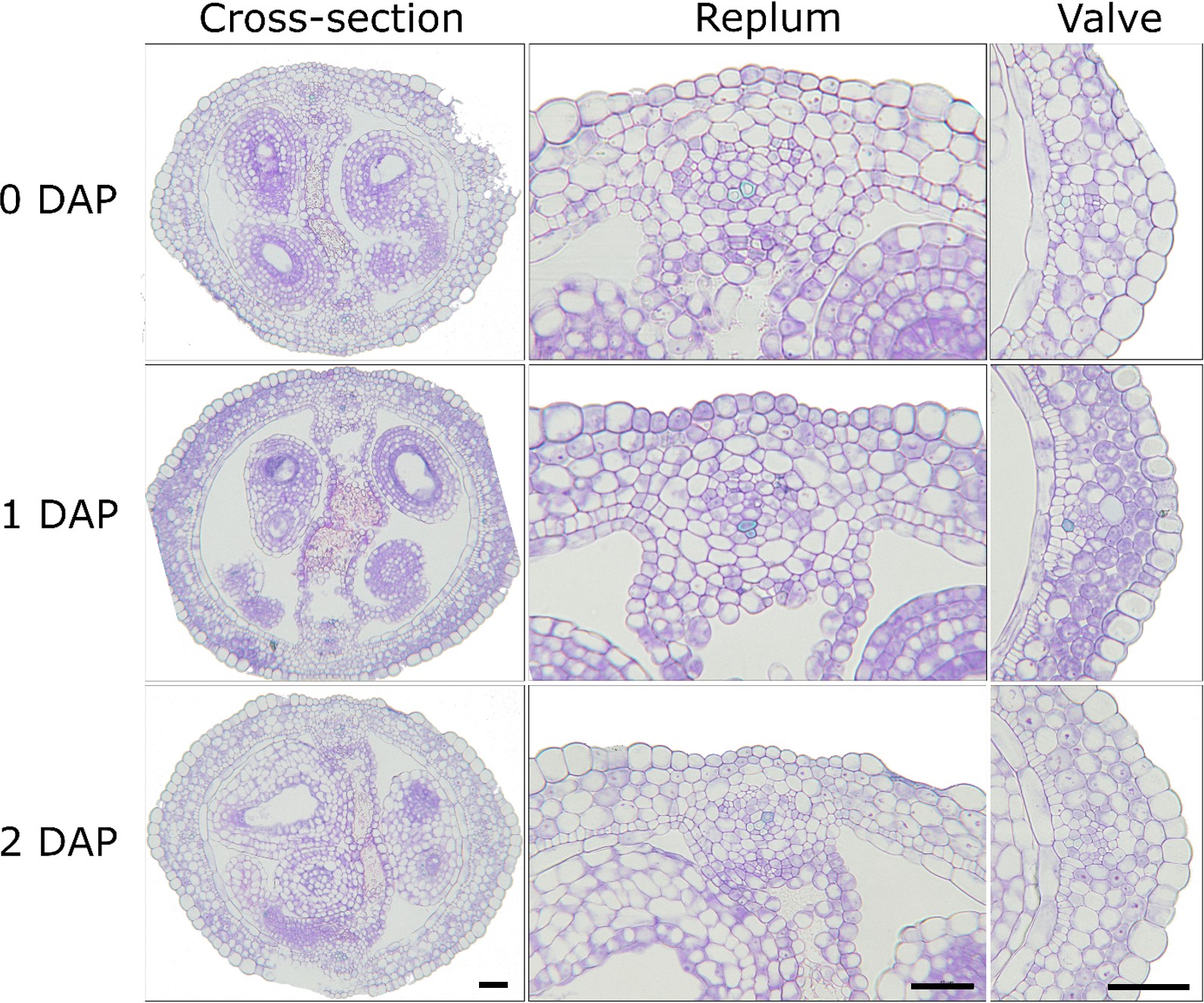


**Supplementary Figure 1. *Arabidopsis thaliana* wild-type silique cross-sections.** Wild-type *Arabidopsis thaliana* ecotype Col-0 at 0-2 days after pollination (DAP) depicting whole silique cross-sections, replum and valve regions. Scale bars = 50 µm for each of the images in the panel.


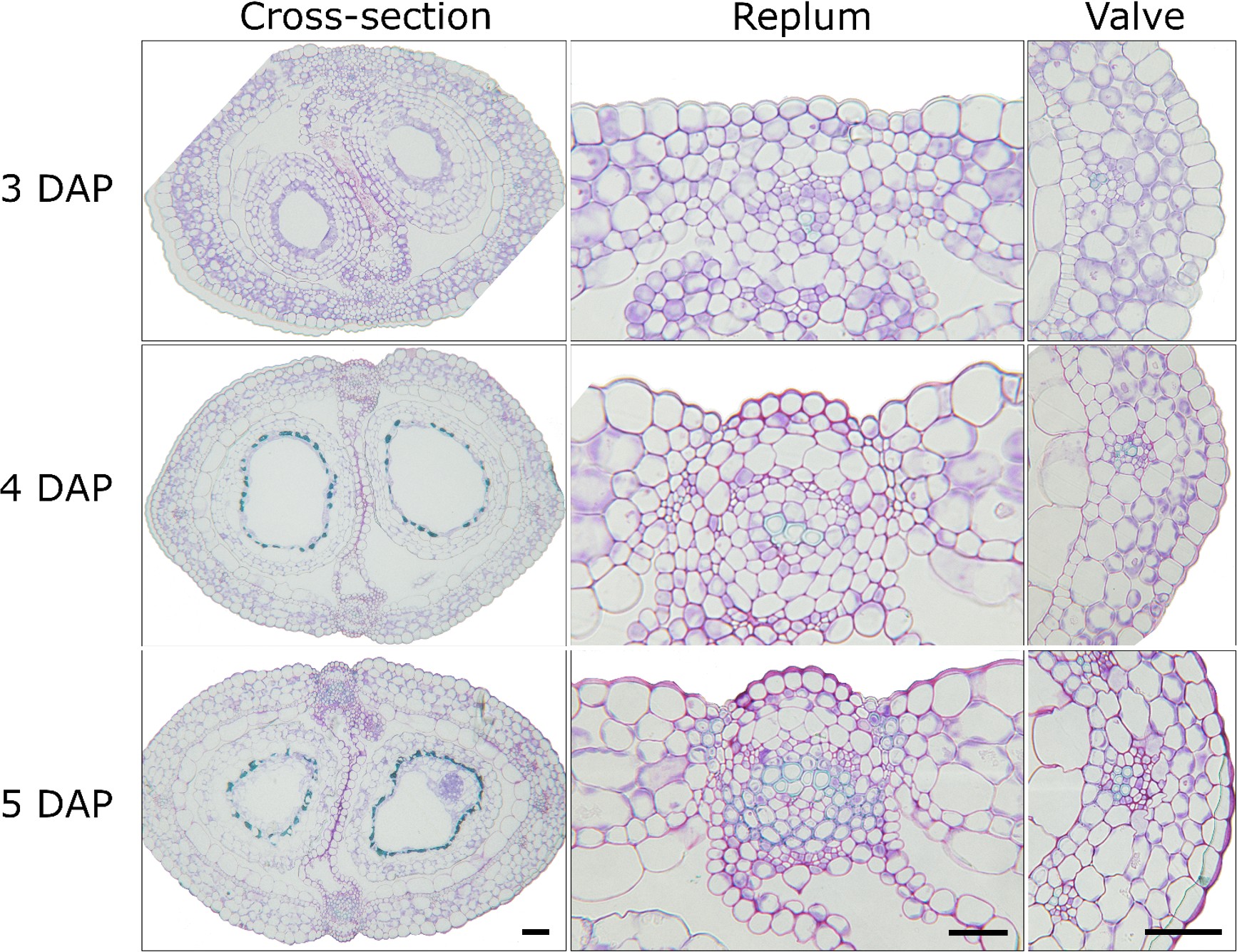


**Supplementary Figure 2. *Arabidopsis thaliana* wild-type silique cross-sections.** Wild-type *Arabidopsis thaliana* ecotype Col-0 at 3-5 days after pollination (DAP) depicting whole silique cross-sections, replum and valve regions. Scale bars = 50 µm for each of the images in the panel.


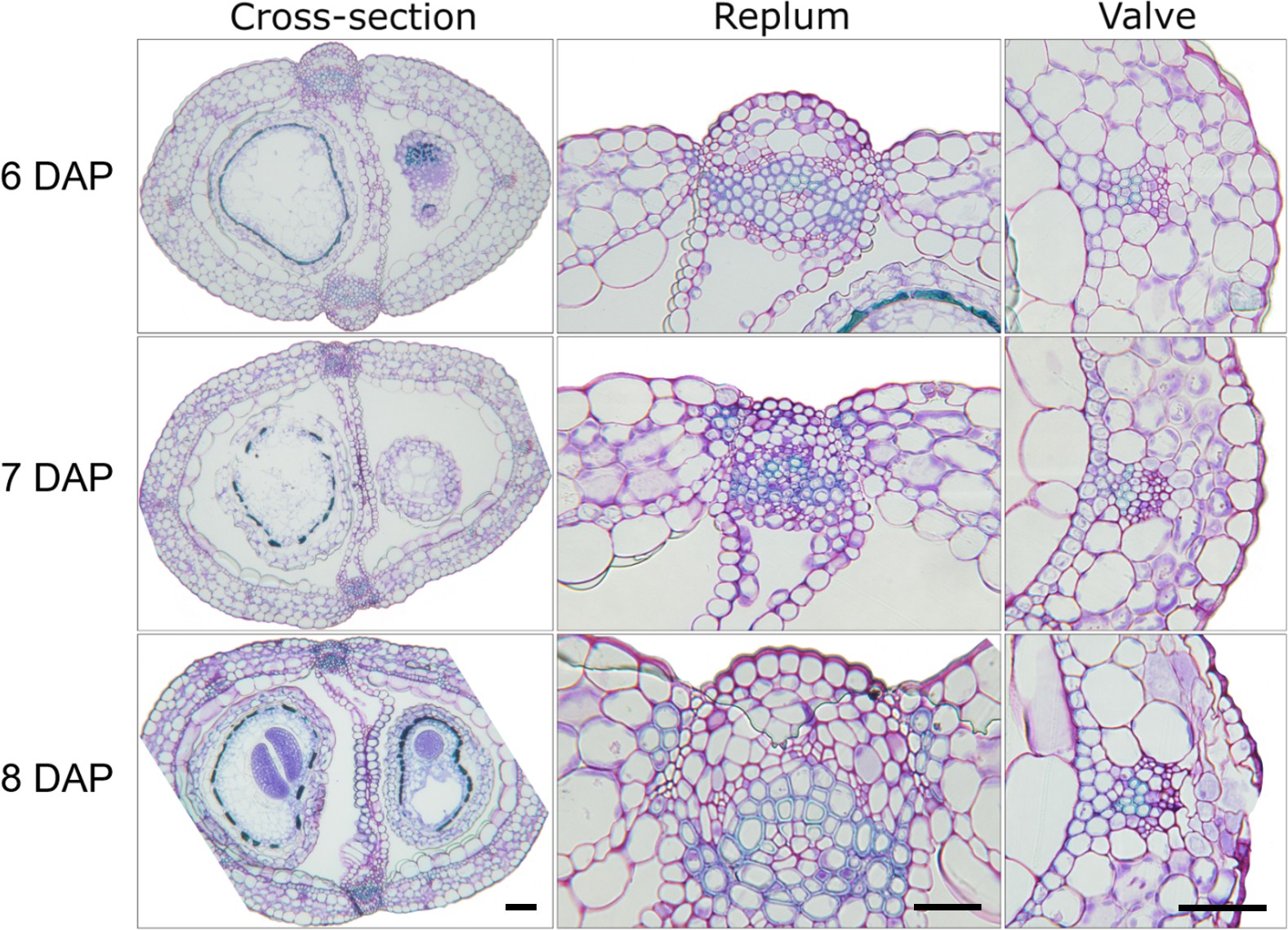


**Supplementary Figure 3. *Arabidopsis thaliana* wild-type silique cross-sections.** Wild-type *Arabidopsis thaliana* ecotype Col-0 at 6-8 days after pollination (DAP) depicting whole silique cross-sections, replum and valve regions. Scale bars = 50 µm for each of the images in the panel.


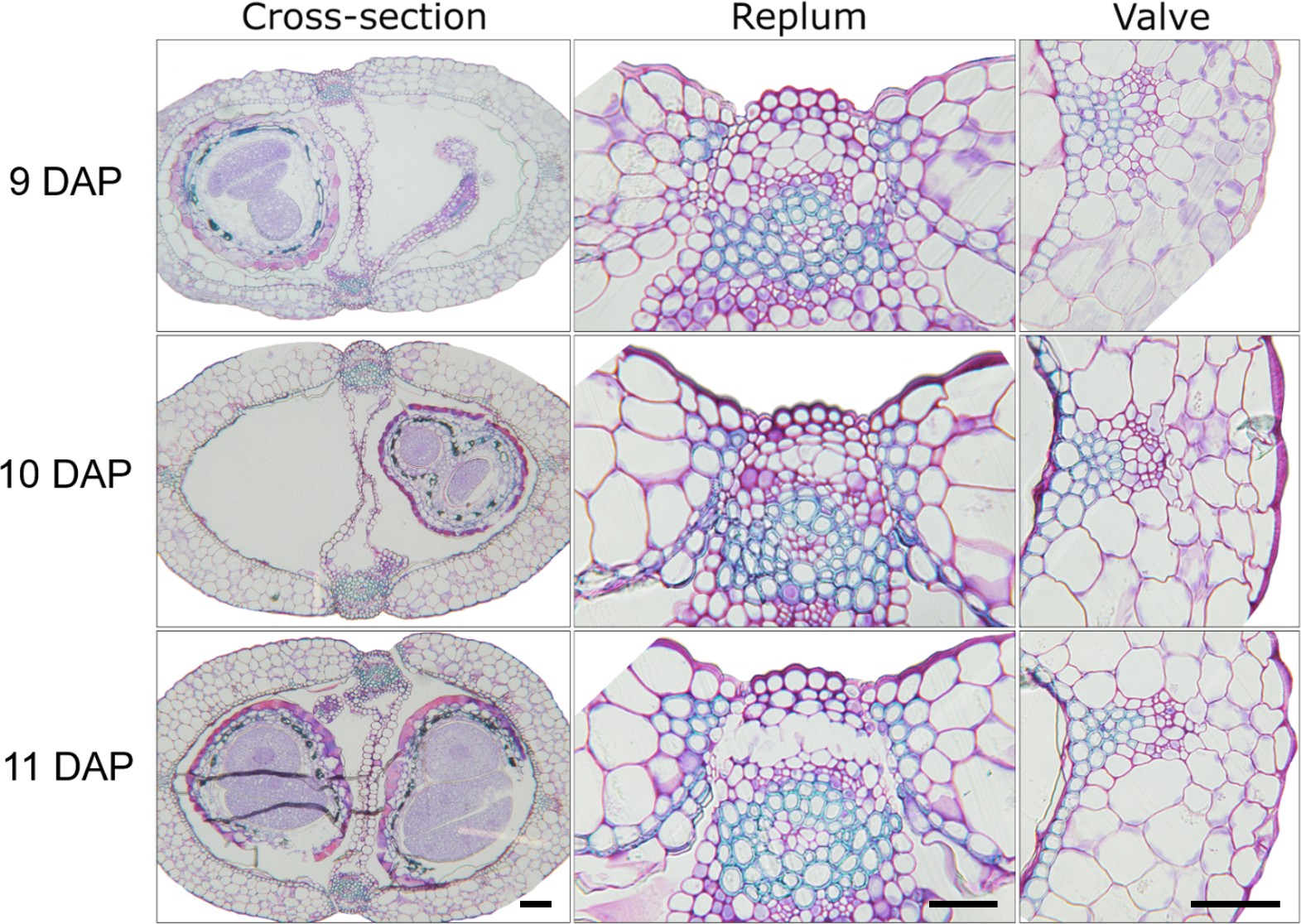


**Supplementary Figure 4. *Arabidopsis thaliana* wild-type silique cross-sections.** Wild-type *Arabidopsis thaliana* ecotype Col-0 at 9-11 days after pollination (DAP) depicting whole silique cross-sections, replum and valve regions. Scale bars = 50 µm for each of the images in the panel.


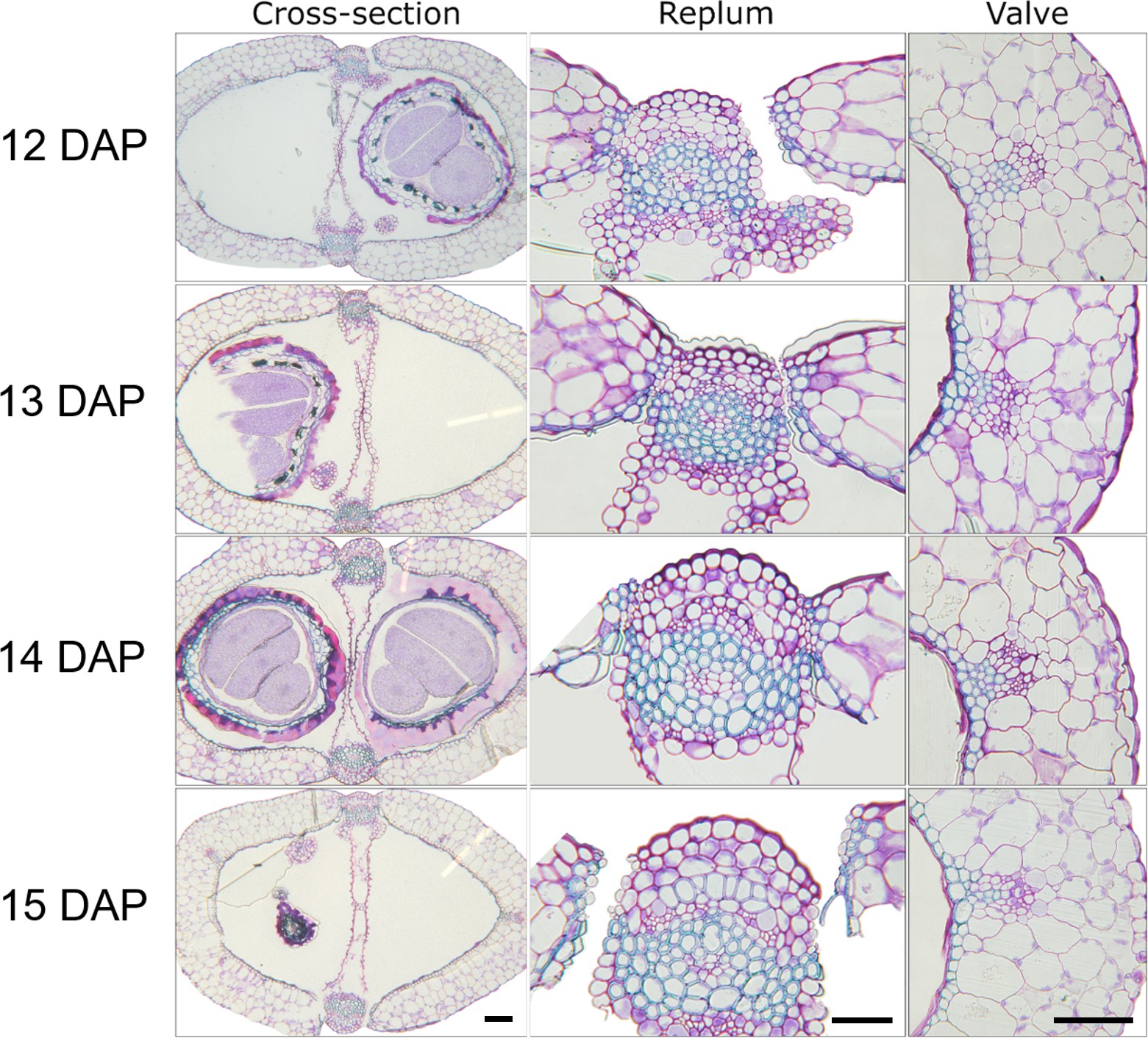


**Supplementary Figure 5. *Arabidopsis thaliana* wild-type silique cross-sections.** Wild-type *Arabidopsis thaliana* ecotype Col-0 at 12-16 days after pollination (DAP) depicting whole silique cross-sections, replum and valve regions. Scale bars = 50 µm for each of the images in the panel.
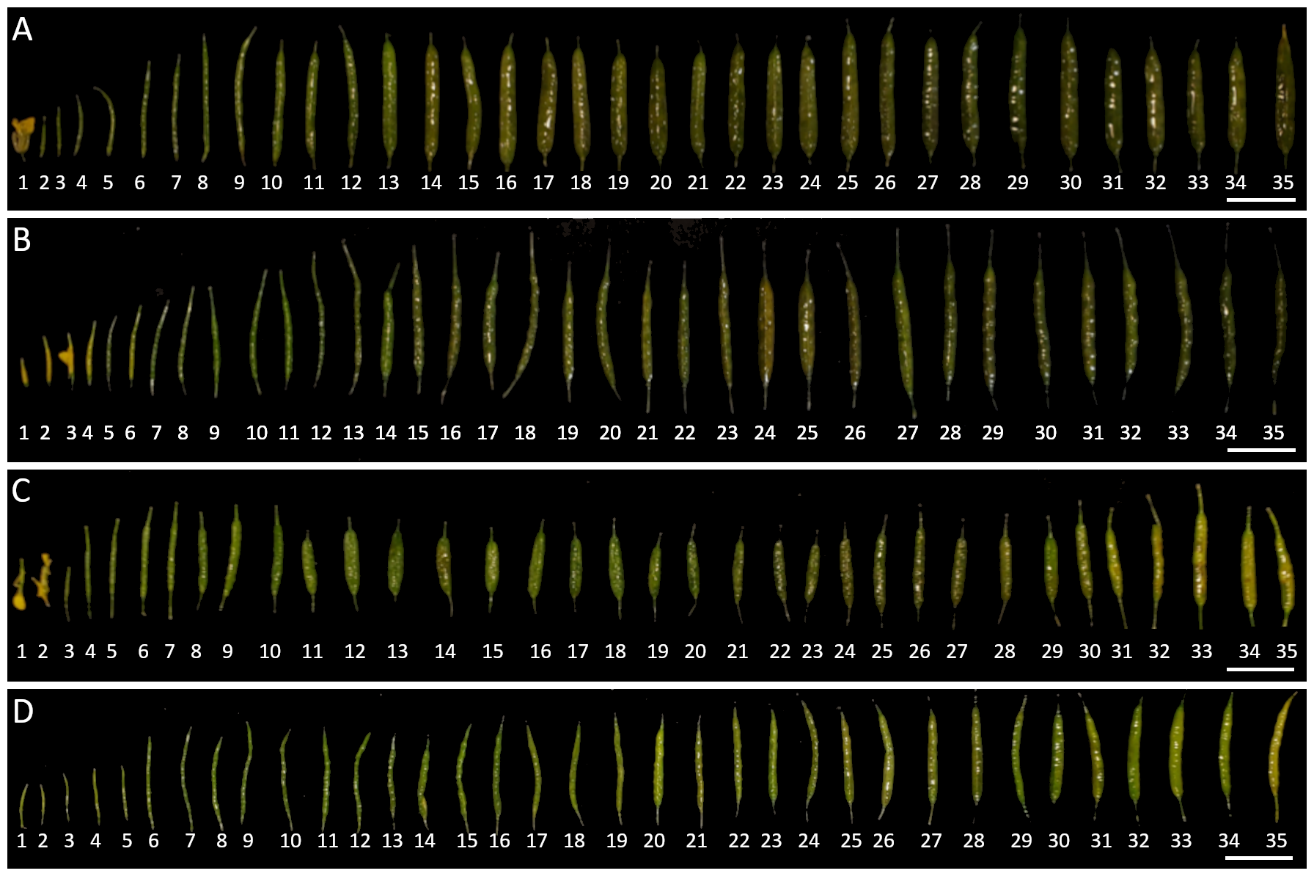


**Supplementary Figure 6. Temporal development of whole Brassicaceae siliques.** Brassicaceae whole siliques from 1 to 35 days after pollination (DAP) which include, *Brassica carinata* (A), *Brassica nigra* (B), *Brassica rapa* (C), and *Brassica juncea* (D). Scale bars = 3 cm.
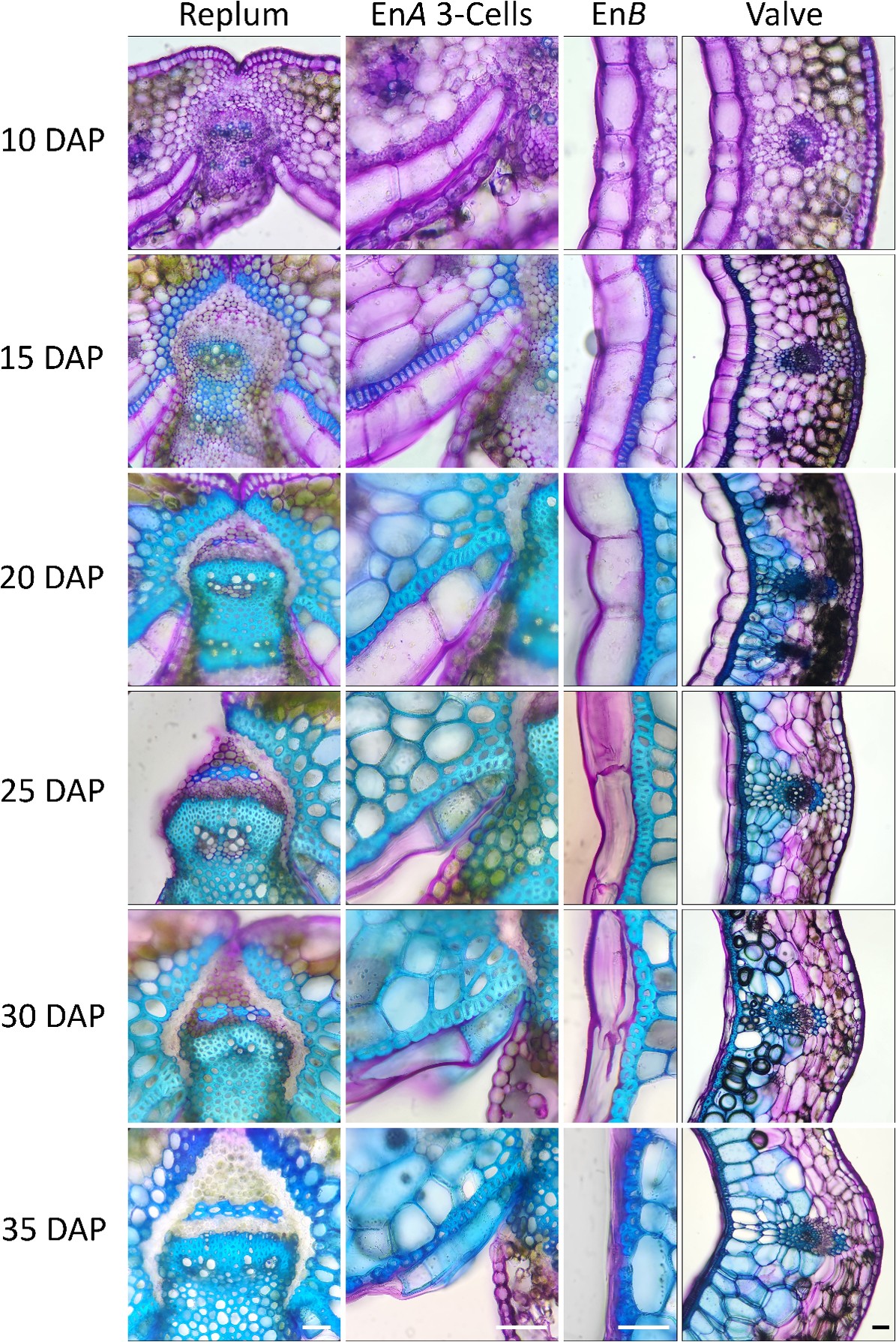


**Supplementary Figure 7. Spatial and temporal ligniﬁcation of *Brassica napus* siliques.** Whole silique cross-sectional images 10, 15, 20, 25, 30, 35 days after pollination (DAP) depicting the replum, endocarp *a* 3- Cells, En*B*, and valve spatial and temporal lignification patterning. Scale bars = 25 µm for each of the images in the panel.
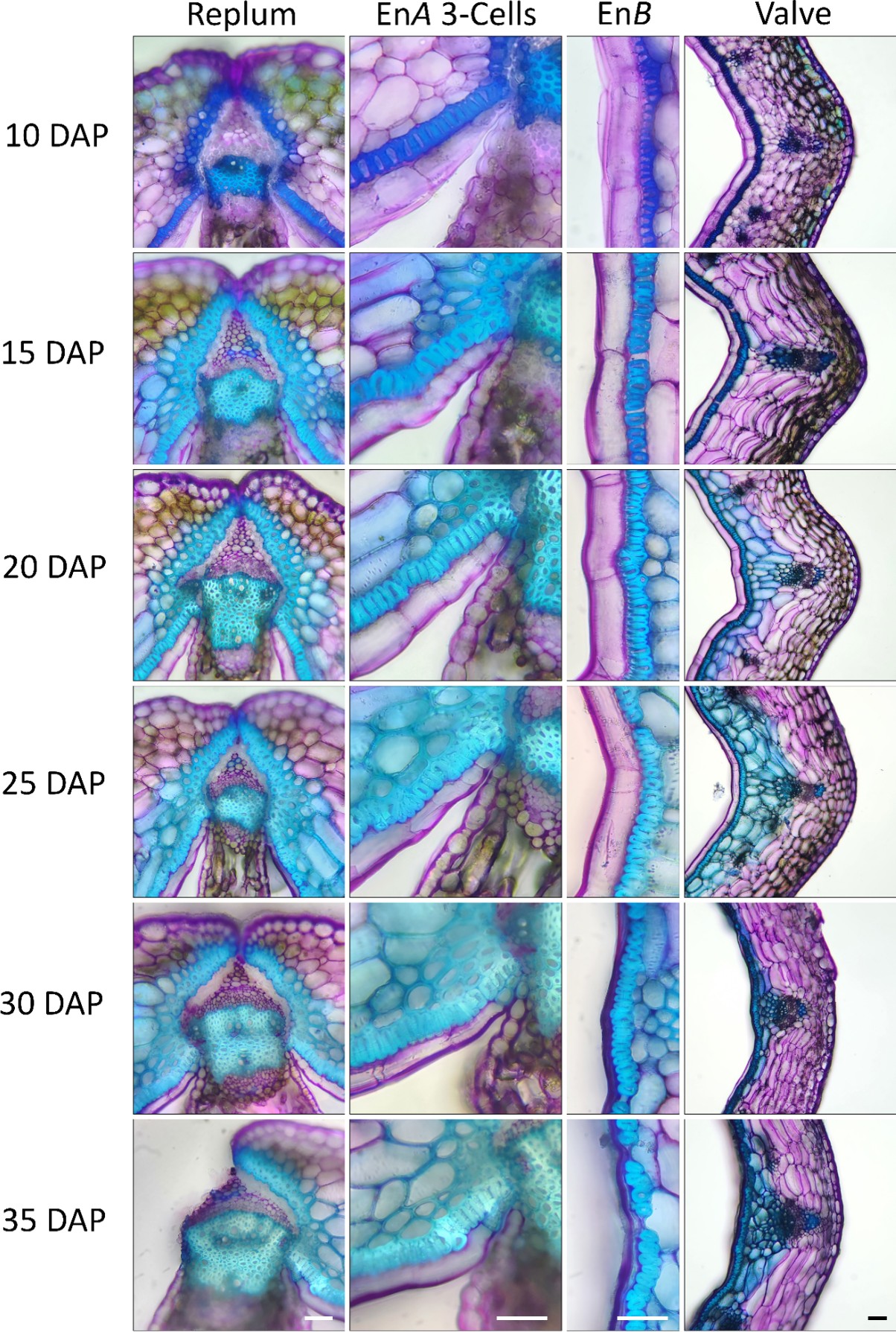


**Supplementary Figure 8. Spatial and temporal ligniﬁcation of *Brassica juncea* siliques.** Whole silique cross-sectional images 10, 15, 20, 25, 30, 35 days after pollination (DAP) depicting the replum, endocarp *a* 3- Cells, En*B*, and valve spatial and temporal lignification patterning. Scale bars = 25 µm for each of the images in the panel.


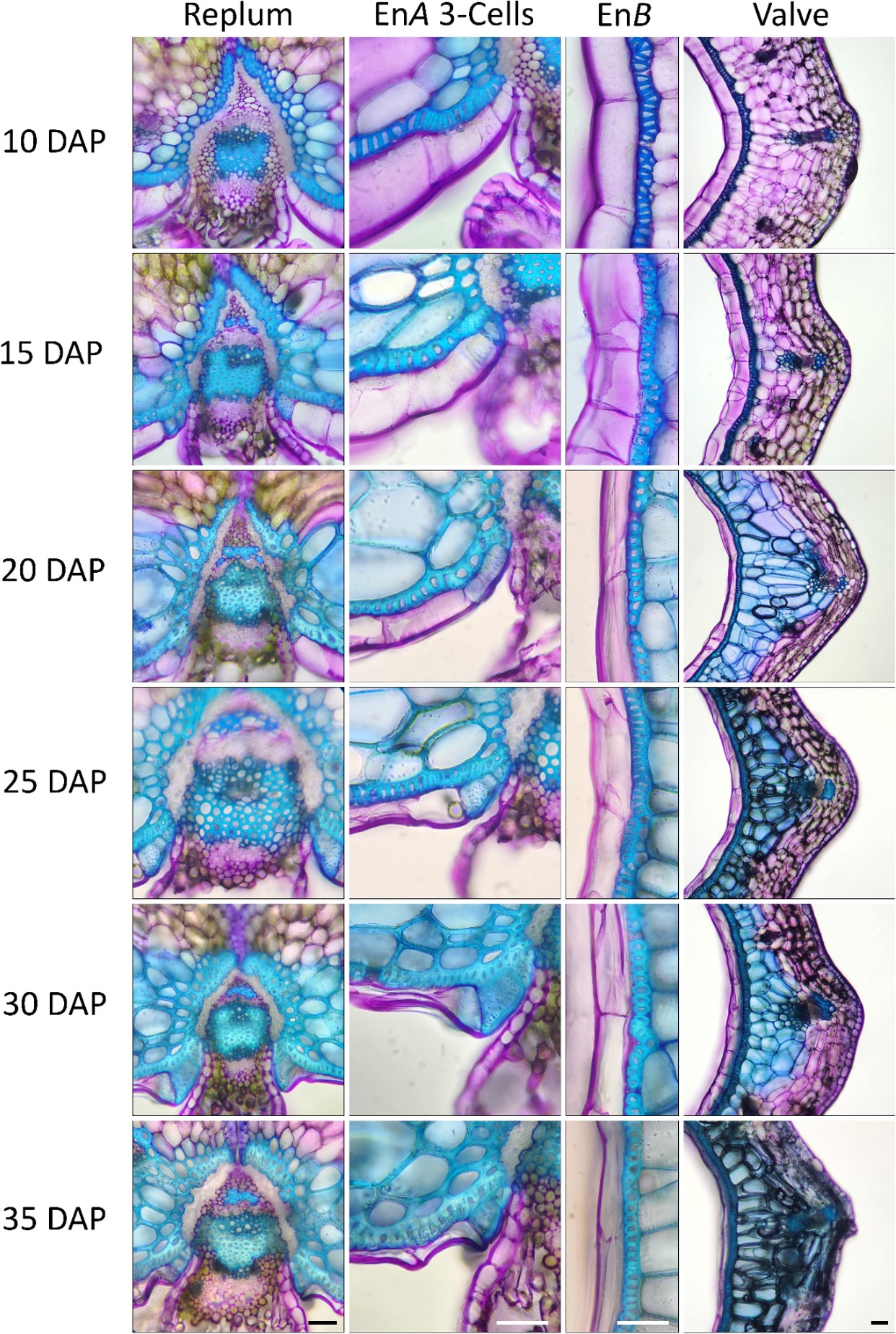


**Supplementary Figure 9. Spatial and temporal ligniﬁcation of *Brassica rapa* siliques.** Whole silique cross-sectional images 10, 15, 20, 25, 30, 35 days after pollination (DAP) depicting the replum, endocarp *a* 3- Cells, En*B*, and valve spatial and temporal lignification patterning. Scale bars = 25 µm for each of the images in the panel.


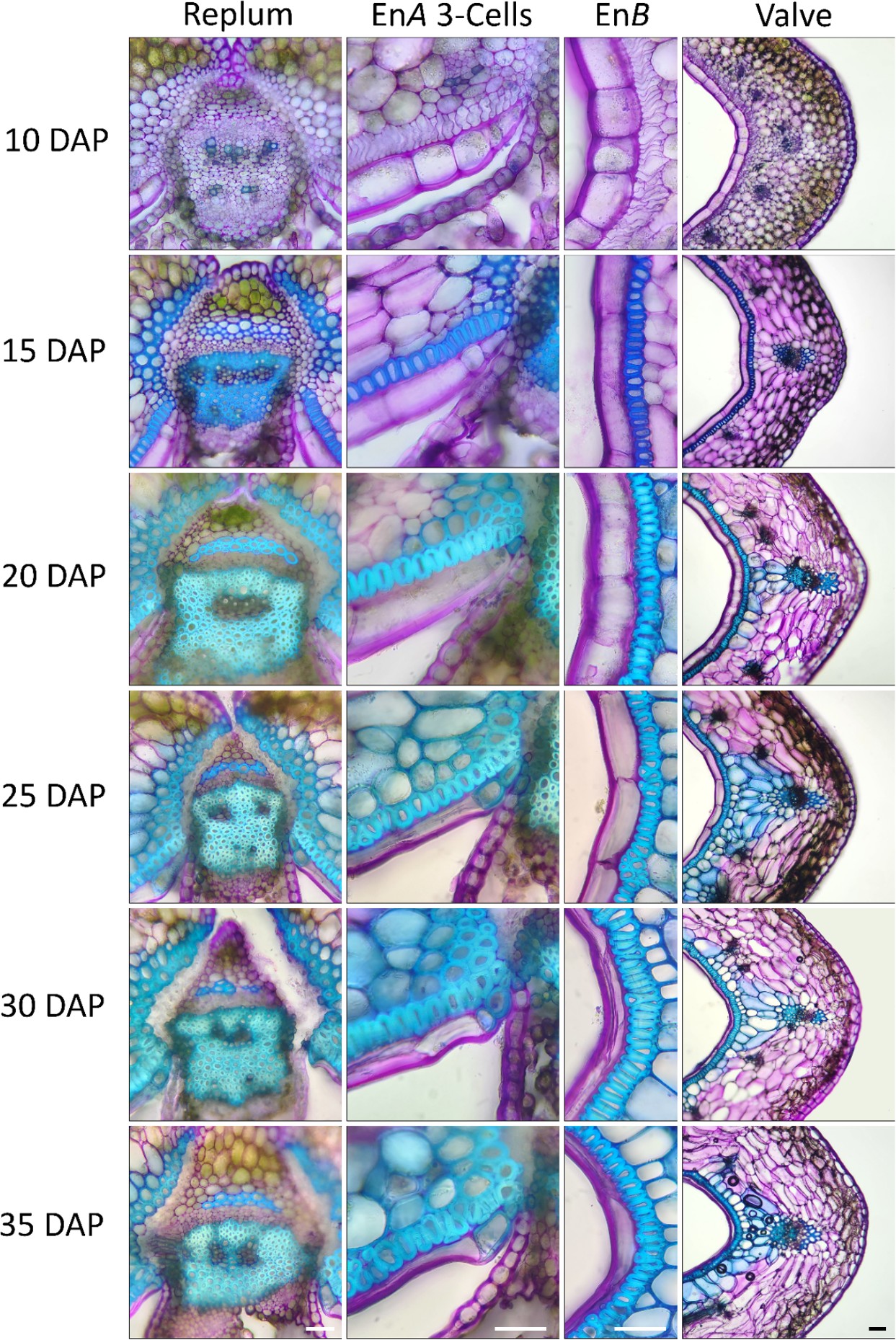


**Supplementary Figure 10. Spatial and temporal ligniﬁcation of *Brassica carinata* siliques.** Whole silique cross-sectional images 10, 15, 20, 25, 30, 35 days after pollination (DAP) depicting the replum, endocarp *a* 3- Cells, En*B*, and valve spatial and temporal lignification patterning. Scale bars = 25 µm for each of the images in the panel.
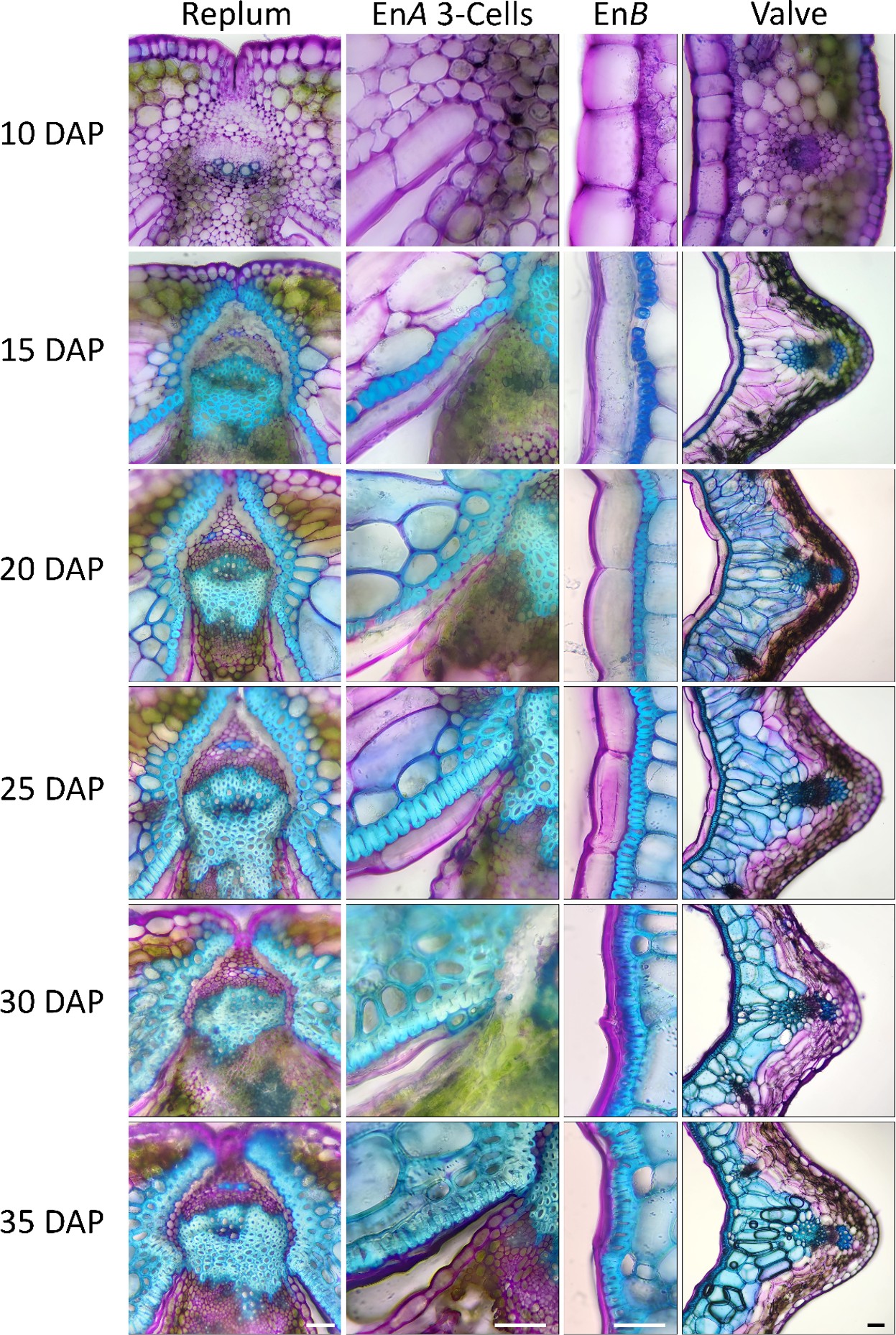


**Supplementary Figure 11. Spatial and temporal ligniﬁcation of *Brassica nigra* siliques.** Whole silique cross-sectional images 10, 15, 20, 25, 30, 35 days after pollination (DAP) depicting the replum, endocarp *a* 3- Cells, En*B*, and valve spatial and temporal lignification patterning. Scale bars = 25 µm for each of the images in the panel.
